# Naïve/Effector CD4 T cell ratio as a useful predictive marker of immune reconstitution in late presenter HIV patients: A multicenter study

**DOI:** 10.1371/journal.pone.0225415

**Published:** 2019-12-23

**Authors:** Veronica Bordoni, Bruno Brando, Pierluca Piselli, Olindo Forini, Federico Enrico Perna, Umberto Atripaldi, Sara Carputo, Federica Garziano, Elisabetta Trento, Giovanna D’Agosto, Alessandra Latini, Manuela Colafigli, Antonio Cristaudo, Alessandra Sacchi, Massimo Andreoni, Gabriella De Carli, Nicoletta Orchi, Sandro Grelli, Arianna Gatti, Carlotta Cerva, Antonella Minutolo, Marina Potestà, Maria Luisa Di Martino, Francesco Ortu, Paola Selva, Laura Del Pup, Irene Guarnori, Patrizia Lorenzini, Giusy Capuano, Andrea Antinori, Chiara Agrati

**Affiliations:** 1 INMI L. Spallanzani IRCCS, Rome, Italy; 2 Blood Transfusion Center, Legnano General Hospital, Legnano, Italy; 3 Department of Clinical Medicine and Surgery, University of Naples Federico II, Naples, Italy; 4 Department of Clinical Biochemistry, Ospedali dei Colli, Naples, Italy; 5 San Gallicano Dermatological Institute IRCCS, Rome, Italy; 6 Clinical Infectious Diseases, Department of System Medicine, University of Rome “Tor Vergata”, Rome, Italy; 7 Clinical Department, INMI L Spallanzani IRCCS, Rome, Italy; 8 Clinical Microbiology and Virology Unit, Department of Experimental Medicine, University of Rome “Tor Vergata”, Rome, Italy; 9 General Pathology and Immunology, Department of Biology, University of Rome “Tor Vergata”, Rome, Italy; 10 Laboratorio di Allergologia e Diagnostica dell'HIV della S.C. Medicina Interna, Azienda Ospedaliero Universitaria Cagliari, Cagliari, Italy; 11 Dipartimento di Medicina, Azienda Ospedaliero Universitaria Cagliari, Cagliari, Cagliari, Italy; 12 Laboratorio Unico Metropolitano (LUM), Azienda USL, Bologna, Italy; 13 UOC Anatomia Patologica, Azienda ULSS2 Marca Trevigiana, Treviso, Italy; 14 Department of Transfusion Medicine and Hematology, Azienda Ospedaliera della Provincia di Lecco, Alessandro Manzoni Hospital, Lecco, Italy; 15 Becton Dickinson Italia, Milan, Italy; Fundación Huésped, ARGENTINA

## Abstract

A significant proportion of HIV-infected patients experiencing a late diagnosis highlights the need to define immunological protocols able to help the clinicians in identifying patients at higher risk for immunological failure. The aim of the study was to evaluate the feasibility of easy cytometric tests in defining the effect of antiretroviral treatment (cART) on immunological homeostasis and in identifying predictive markers of early immune recovery. Chronic HIV infected patients (n = 202) were enrolled in a prospective multicentric study, and their immunological profile was studied before (w0) and after 24 weeks (w24) of antiretroviral treatment (cART) using a standardized flow cytometric panel. Based on CD4 T cell count before treatment, patients were divided in late (LP: CD4 <350/mmc), intermediate (IP: 350/mmc<CD4<500/mmc) and early (EP: CD4 >500/mmc) presenters. In all groups, cART introduction increased CD4 and CD4/CD8 T cell ratio, naïve T cell (CD4 and CD8) and CD127-expressing CD4 T cells. In parallel, cART significantly reduced effector memory T cells (CD4 and CD8) and T cell activation (CD38+CD8 and CD95+CD4 T cells). Moreover, the frequency of Naïve and Effector CD4 T cells before treatment correlated with several immune parameters key associated with the pathogenesis of HIV, thus mirroring the health of immune system. Interestingly, we identified the Naïve/Effector CD4 T cell ratio (N/EM) at w0 as a marker able to predict early immune recovery. Specifically, in LP, N/EM ratio was significantly higher in immunological responder patients (CD4>500/mmc at w24) when compared to immunological non responder (CD4 T cells <500/mmc at w24). Finally, a multivariate analysis indicates that after 24w patients with N/EM ratio higher than 1.86 at w0 recovered 96 CD4 T cells more than those with N/EM ratio lower than 0.46. Altogether, our data define an easy protocol able to define reliable immunological markers useful for the characterization of immune profile in viremic HIV patients and identify the naïve/effector CD4 T cell ratio as a new tool able to predict an early immune reconstitution potential.

## Introduction

The introduction of combined antiretroviral therapy (cART) has deeply changed the management of HIV infection with a reduction of morbidity and mortality of HIV-1–infected individuals. Nevertheless, despite effective control of HIV replication, some individuals experienced limited recovery of CD4+ T cell counts [1,2]. These immunological non responder patients present an higher risk for clinical progression than patients in whom CD4 T cell count is restored [3–5]. Although the detailed pathological mechanisms responsible for immunological failure are not completely defined, several parameters have been proposed as strongly associated to an inadequate immune restoration. In particular, age [4,6] nadir CD4+ T cell count [7,8], low CD4/CD8 T cell ratio [9–12], duration of HIV-1 infection [4,6], CD4 and CD8 T cell activation [1], inflammation and microbial translocation [1] have been associated with a failure of immune recovery (reviewed in 2). Moreover, a decrease in circulating naïve CD4 and CD8 T cells [1,13] and a reduction in the response to IL-7 homeostatic stimulation [14–17] have been reported in immunological non responder patients. Several other factors have been found correlated with immunological response, such as polyfunctional HIV specific CD8 T cell subset [18] and microbiota profile, [19] but they are not easily used in a routine diagnostic scale.

The CD4 and CD8 T cell quantification is easily performed by well-standardized flow cytometry protocols: CD4, CD8 and CD4/CD8 T cell ratio are currently used in monitoring HIV infection before and after treatment and represent the most important markers of immune recovery. A significant HIV population experiences a late diagnosis (with a low number of CD4 T cells) and represents a group of patients needing particular attention and a more detailed immune monitoring. In these patients, the definition of predictive markers of immune recovery could help the clinicians in identifying patients at higher risk for an immunological failure.

The standardization of flow cytometric analysis is a key issue in the context of immune monitoring. A standardized 8-color flow cytometry panel, able to simultaneously define activation, maturation and senescence of CD4 and CD8 T lymphocytes in HIV-infected individuals, has been tested in a cross-sectional [20] and in a longitudinal study [21], showing the persistence of immunological alterations despite long term effective cART.

We performed a longitudinal multicentric study aimed to evaluate the feasibility of easy cytometric tests in defining the effect of cART on immunological profile and in identifying predictive markers of early immune recovery.

## Materials and methods

### Patient population

Chronic newly diagnosed, therapy naïve HIV infected subjects were enrolled in this study in different Italian clinical centers: i) INMI L Spallanzani of Rome, ii) San Gallicano Dermatological Institute IRCCS iii) University of Tor Vergata, iv) ASL Treviso, v) Asl, Legnano, vi) Hospital Cotugno of Naples, vii) Policlinico, Cagliari, viii) Ospedale Manzoni, Lecco and ix) Policlinico S.Orsola, Bologna. The study was approved by INMI Ethical committees, and a written signed informed consent was obtained from all participants (approval no. 78 dated November 21, 2013). Patients general characteristics were anonymously abstracted from clinical charts and are summarized in Table 1. Enrolled patients were sampled before (w0) and after 24 weeks (w24) from cART initiation.

**Table 1:** Clinical features of HIV patients.

		HIV patients (N = 202)
**Gender (M/F)**	N°	184/18
**Age (years)**	Median (IQR)	37 (30–45)
**CD4 at baseline (cells/mmc)**	Median (IQR)	358 (190–511)
**HIV-RNA at baseline (log)**	Median (IQR)	4.8 (4.3–5.3)
**HIV-RNA at w24**	N° of patients (%)	
Target non-detected (<40 cp/ml)		46 (22.8)
Detected <40 cp/ml		116 (57.4)
Detected ≥40cp/ml		40 (19.8)
**Type of ARV regimen**	N° of patients (%)	
INSTI		45 (22.3)
NNRTI		63 (31.2)
PI/b		42 (20.8)
Other or unknown		52 (25.7)

Patients with CD4>500/mmc at w24 were defined as immunological responders (IR), while patients with CD4<500/mmc at w24 were defined as low immunological responder (LR).

### Flow cytometry

Whole blood samples were stained using preconfigured lyophilized reagent tubes (BD Lyotube TM 8-color CD4 and CD8 bundle, (BD Biosciences San Jose, CA, USA): CD4 Lyotube: CD95 FITC/ CCR7 PEl CD3 PerCP- Cy 5.5/ CD25 PE-Cy7/ CD127 Alexa Fluor 647/ CD45 APC-H7/ CD4 V500/ CD45RA V450; clones: DX2/ 150503/ SK7/ 2A3/ HIL-7R-M-21/ 2D1/ SK3/ HIlOO, respectively. CD8 Lyotube: CD38 FITC/ CCR7 PEl CD3 PerCPCy 5.5/ CD69 PE-Cy7/ CDl27 Alexa Fluor 647/ CD45 APC-H7/ CD8 V500/ CD45RA V450; clones: HB7/ 150503/ SK7/ L78/ HIL-7R-M-21/ 2D1/ SK1/ HIlOO, respectively. Lyotubes were rehydrated with 100 ul of blood and incubated for 5 minutes before mixing. After an additional 30 minutes of incubation at room temperature, samples were lysed with 2 mL of lysing buffer (BD PharmLyse) at 4°C in the dark. After washing, samples were acquired within 1 hour with a FACSCanto II flow cytometer (BD Biosciences) equipped with blue (488 nm), red (633 nm) and violet (405 nm) lasers. Instrumental sensitivity was evaluated and monitored over time using the BDTM Cytometer Setting &Tracking system. The 8-Color Lyotube kit included single-color lyophilized control tubes for each reagent used in this study, Antibody capture beads (CompBeads, BD Biosciences) were used for single-color compensation controls for each reagent. A total of 200,000 events were collected per sample. The analysis of cytometric data was performed using BD FACSDiva software version 6.1.3 (BD Biosciences).

In order to achieve consistent and comparable data over the time and across the different sites, all the BD FACSCanto II cytometers are standardized using BD CS&T beads, creating an Application Setting and defining Target Values (TG). The optimal PMT voltage for each fluorescence channel was first determined on the BD FACSCanto II sited in INMI L.Spallanzani, using the PMT minimal values provided by the Diva software. Using a standardized voltage set, BD CS&T and Rainbow beads were run on, without compensation, to define TG. The median fluorescence intensity (MFI) of the seventh peak of Rainbow and MFI of the brightest peak of BD CS&T beads were recorded in all eight fluorescence channels. These TG were directly used to setup PMT voltages in the rest of BD FACSCanto II instruments. Before any acquisition, PMT voltage were updated using Application Setting and maintenance of TG are verified running beads. The gating strategy used in this work was previously described [20]. Briefly, first, lymphocytes were gated based on FSC-A and SSC-A to exclude debris and on FSC-A and FSC-W to remove doublets, followed by CD45+ gating to identify the lymphocyte population. CD4+ and CD8+ T cells are further selected among CD3+ population. CD4 and CD8 T cell maturational subsets were defined as: Naïve (NA; CD45RA+CCR7+), Central Memory (CM; CD45RA-CCR7+), Effector Memory (EM; CD45RA-CCR7-), Terminal Effector Memory (TEMRA; CD45RA+CCR7-). Regulatory T cells were identified as CD4+CD25highCD127-. The expression levels of CD95, CD25 and CD127 molecules were defined in all CD4+ T-cell subsets; similarly, expression levels of CD38, CD69 and CD127 molecules were determined in all CD8+ T-cell subsets.

### Statistical analysis

GraphPad Prism version 5.0a (GraphPad Software, USA) and STATA 15.1 were used to perform statistical analysis and graphs. Kruskal Wallis with Dunns correction test was used to compare groups and a non parametric Wilcoxon test was used to compare variables throughout follow-up. The correlations were performed using the non parametric Spearman test. A p-value less than 0.05 was considered statistically significant. Linear regression was used to investigate the independent association between the recovery of CD4 T cell at 6 months from baseline with baseline value of EM CD4 T cells. At multivariable model the association between CD4 change and N/EM ratio was adjusted for *a priori* set of covariates (age, CD4 cell count and CD4/CD8 ratio at cART initiation).

## Results

### Effect of cART introduction on CD4 and CD8 T cells homeostasis

**Fig 1:**
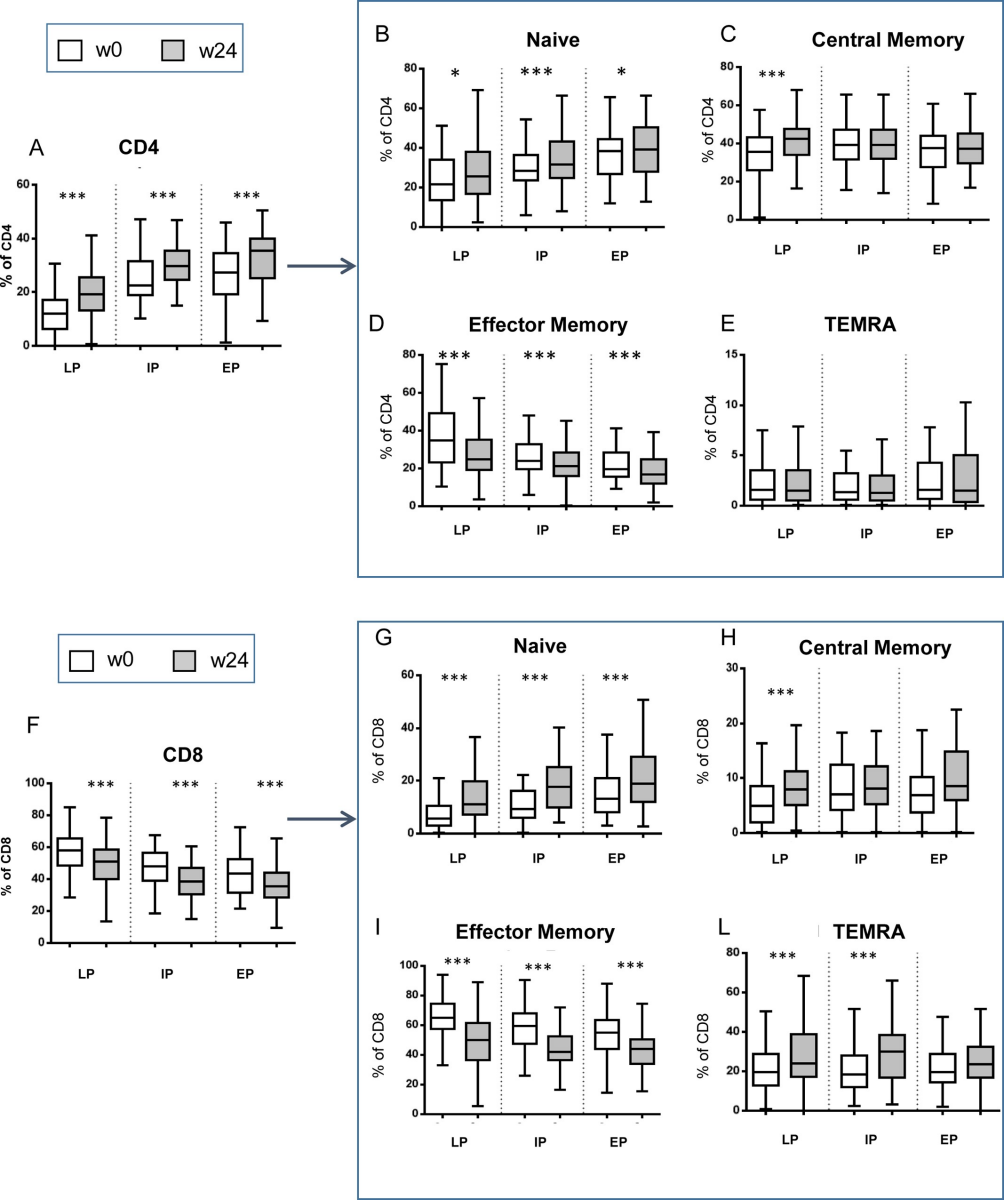
Impact of cART on CD4 and CD8 T cell differentiation profile. The differentiation profile of CD4 **(A-E)** and CD8 **(F-L)** T-cells was evaluated by flow cytometry before (wo, white bars) and after (w24, grey bars) therapy. The differentiation subsets of CD4 and CD8 T cells were defined as follow: **B, G:** Naive (CCR7+CD45RA+); **C, H:** Central Memory (CCR7+CD45RA-); **D, I:** Effector Memory (CCR7-CD45RA-); **E, L:** Terminally Differentiated (CCR7-CD45RA+). HIV patients were divided in three groups [late (LP), intermediate (IP) and early (EP) presenters] on the basis of their CD4 T cell counts at w0. Results are shown as Box and Whiskers graphs. The parametric Wilcoxon test was used, and p<0.05 was considered significant. *: p<0.05; ***: p<0.001.

To assess the effect of cART introduction on the homeostasis of the CD4 and CD8 T cells, a flow cytometric analysis was performed. Specifically, the differentiation and activation profile of CD4 and CD8 T cells was analyzed before and after 24 weeks of cART introduction. To discriminate a possible impact of different stages of HIV disease on the immune reconstitution process, the enrolled HIV patients were divided on the basis of CD4 T cell count at w0 in three groups (Table 2): i) late presenters (CD4 T cells ≤350/mmc); ii) intermediate presenters (350/mmc<CD4 T cells <500/mmc) and iii) early presenters (CD4 T cells ≥500/mmc). Baseline HIV-RNA and age were significantly different among the three groups (Table 2). As expected, cART was effective in increasing the frequency of CD4 T cells in all groups (Fig 1A). In particular, an increase of naïve (Fig 1B) and a parallel reduction of effector memory (EM) CD4 T cells (Fig 1D) were observed in all groups of patients. In contrast, an increase of central memory (CM) CD4 T cells was found only in LP patients (Fig 1C). No significant effects of cART was observed on terminally differentiated (TEMRA) CD4 T cells (Fig 1E). A similar profile was obtained when looking at CD8 T cells (Fig 1 F-L). Indeed, cART was able to significantly reduce CD8 T cell frequency (Fig 1F) in all groups with an increase of naïve (Fig 1G) and a parallel reduction of EM CD8 T cells (Fig 1I). As for CD4 T cells, the ability of cART to increase CM subset was limited to LP patients (Fig 1H). Finally, an increase of TEMRA CD8 T cells was observed in IP and in LP patients (Fig 1L).

**Table 2:** HIV patients were divided in three groups based on their CD4 T cell count before treatment. Patients were defined late (LP), intermediate (IP) and early (EP) presenters.

	LP CD4<350 (N = 98)	IP 350<CD4≤500 (N = 51)	EP CD4>500 (N = 53)	
	N° or median (IQR)			*p*
**Gender (M/F)**	86/12	49/2	49/4	0.18
**Age (years)**	41 (31–47)	32 (27–29)	36 (31–43)	***0*.*001***
**CD4 at baseline (cell/mmc)**	189 (75–266)	433 (395–468)	630 (560–728)	*n*.*a*.
**HIV-RNA at baseline (log)**	5.0 (4.5–5.5)	4.8 (4.2–5.2)	4.6 (4.2–4.9)	***0*.*002***

**Fig 2:**
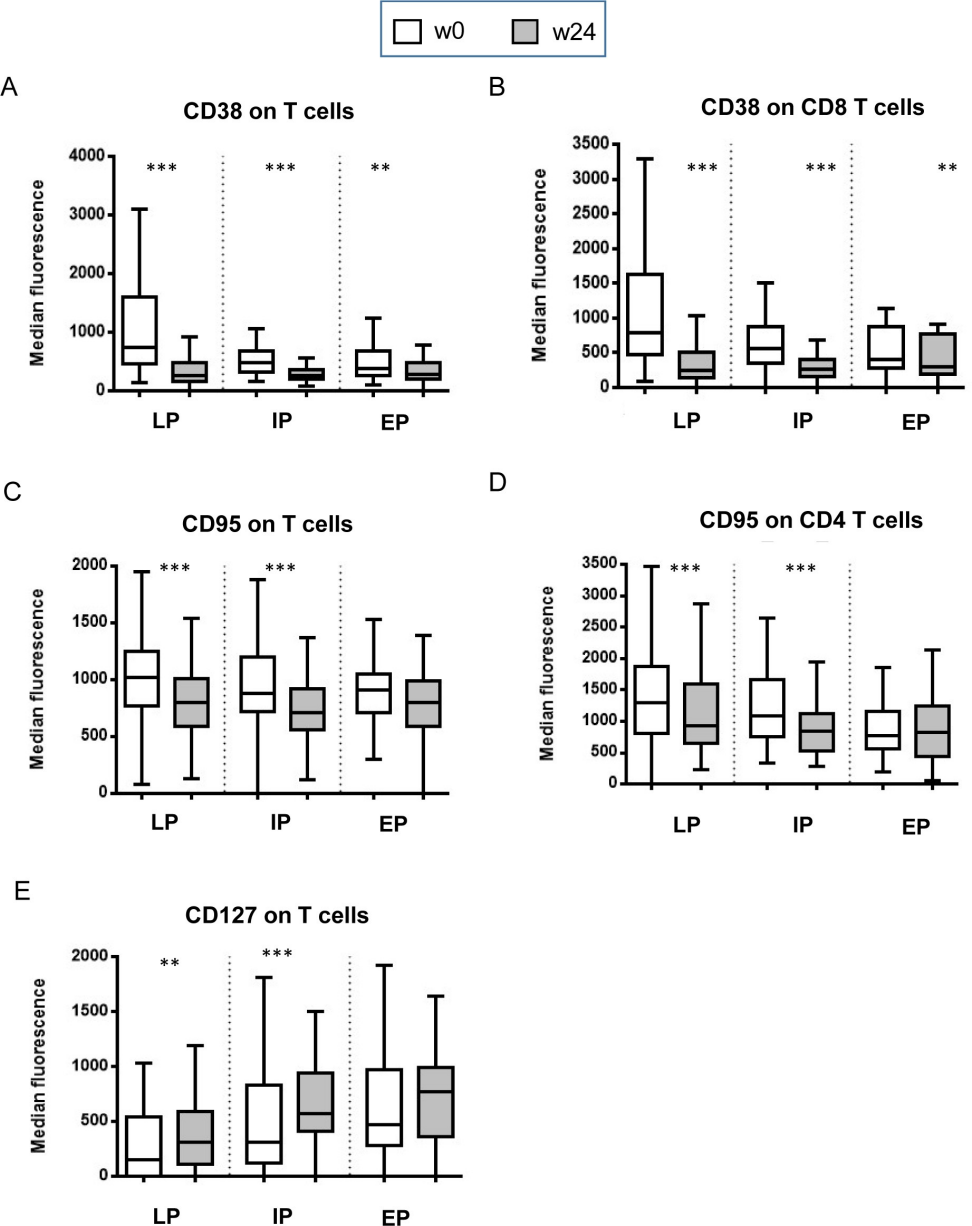
Impact of cART on T cell activation markers. The activation and early marker expression was evaluated by flow cytometry before (wo, white bars) and after (w24, grey bars) therapy. The expression of CD38 was evaluated on T cells **(A)** and on CD8 T cells **(B).** The expression of CD95 was evaluated on T cells **(C)** and on CD4 T cells **(D**). The expression of CD127 was evaluated on T cells **(E).** Results are shown as median of fluorescence intensity (MFI) using Box and Whiskers graphs. The parametric Wilcoxon test was used and p<0.05 was considered significant. **: p<0.01; ***: p<0.001.

Therefore, the impact of cART on the activation profile and on the expression of early T cell marker was evaluated by analyzing CD38 on CD8 T cells and CD95 and CD127 on CD4 T cells (Fig 2). In all groups of HIV patients, cART reduced the expression of CD38 on CD3 T cells (Fig 2A) and on CD8 T cell subset (Fig 2B). Accordingly, a reduction of CD95 expression on CD3 T cells (Fig 2C) and on CD4 T cells (Fig 2D), was observed but was limited to IP and LP patients. In the same groups of patients, an increase of CD127 expression on T cells was also observed (Fig 2E). No significant differences were observed for CD69 and CD127 on CD8 T cells (data not shown). Finally, the frequency of stem cell memory (SCM, defined as CD45RA+CD95+CCR7+) was lower in LP than in EP (p<0.02), confirming an impact of SCM depletion in the disease progression [22].

### Baseline CD4 T cell differentiation strongly correlates with immunological parameters of activation/dysfunction

**Fig 3:**
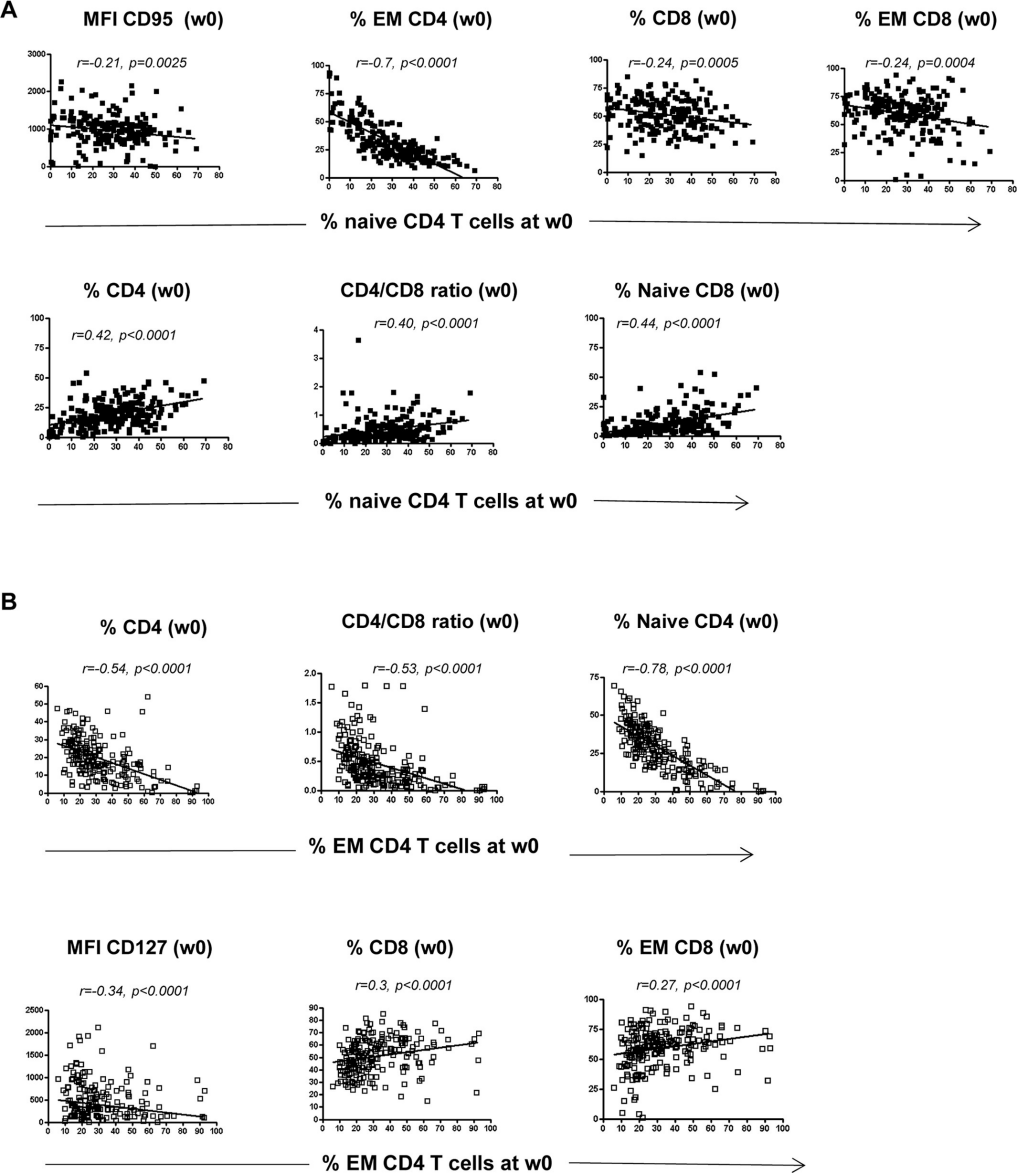
**Baseline frequency (w0) of Naive CD4 T cells (A) and EM CD4 T cells (B) correlates with several baseline immune parameters.** The correlation analysis was performed by Spearman test and a p<0.05 was considered significant.

**Fig 4:**
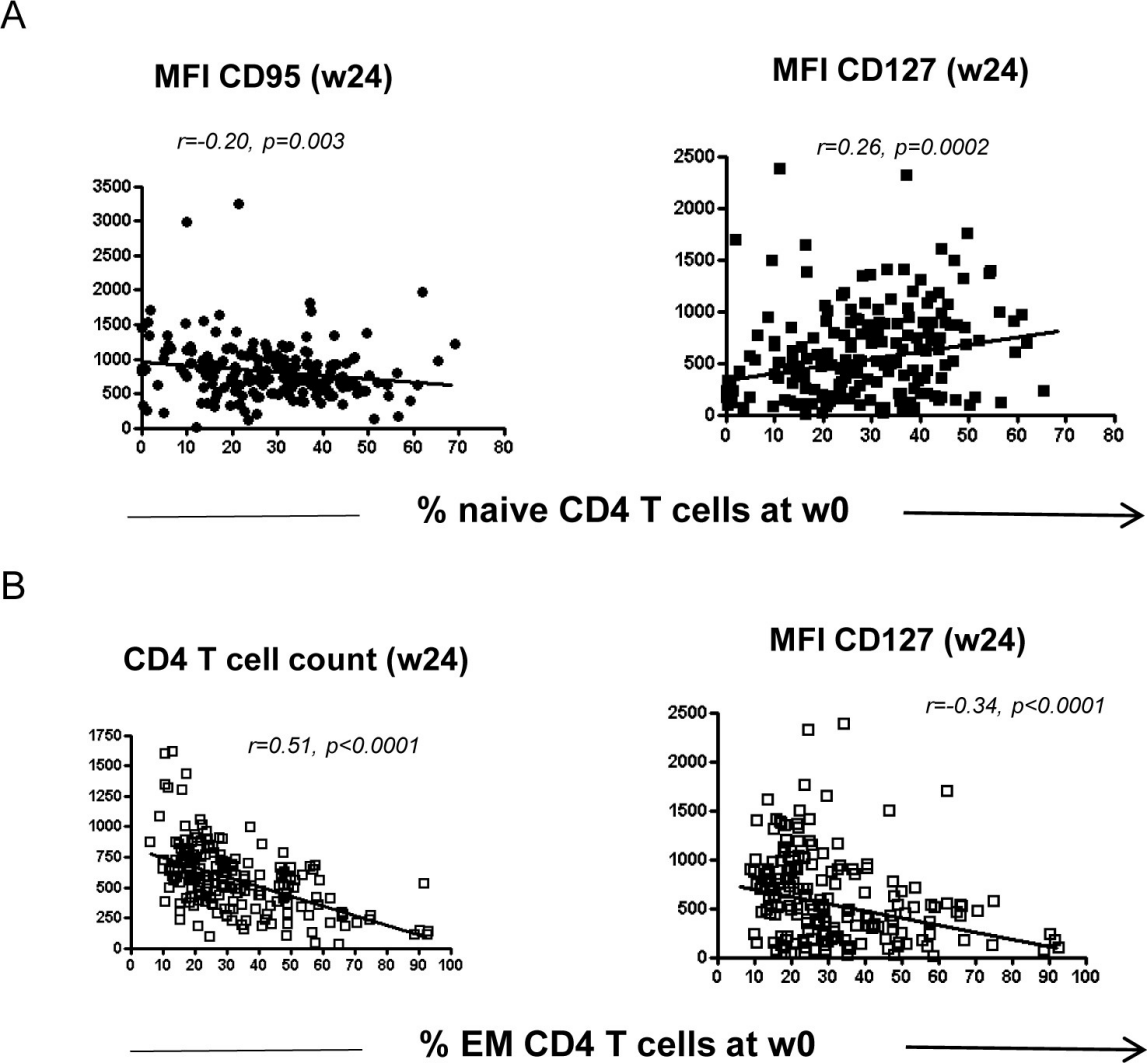
**Baseline frequency (w0) of naive CD4 T cells (A) and EM CD4 T cells (B) correlate with several immune parameters after treatment (w24).** The correlation analysis was performed by Spearman test and a p<0.05 was considered significant.

T cell differentiation is tightly regulated by the microenvironment and can represent a marker of immune homeostasis. We therefore wondered possible associations between the frequency of T cell subsets (naïve and effector CD4 T cells) and other immunological parameters known to be associated with the pathogenesis of HIV disease. Before treatment, the frequency of CD4 naïve T cells was correlated with several other baseline (w0) parameters (Fig 3). Specifically, the frequency of CD4 naïve T cells was inversely correlated with the expression of immune markers associated to pathogenesis (CD95 expression on T cells, EM CD4 T cells, CD8 T cells and EM CD8 T cells), and directly correlated with the expression of beneficial markers (CD4 T cell count, CD4/CD8 T cell ratio and naïve CD8 T cells frequency). On the other hand, the frequency of EM CD4 T cells presented a completely opposite behavior (Fig 3). They were inversely correlated with the expression of beneficial markers (CD4 T cells, CD4/CD8 T cell ratio, naïve CD4 T cells and CD127 on T cells) and directly correlated with pathogenetic markers (CD8 T cells, EM CD8 T cells). Moreover, the frequency of naïve CD4 T cells before treatment was also correlated with immunological parameters after treatment (w24) (Fig 4). Specifically, the frequency of naïve CD4 T cells before treatment was negatively associated with the expression of CD95 on T cells at w24 and positively correlated with the expression of CD127 on T cells at w24. On the other hand, the frequency of EM CD4 T cells was negatively correlated with CD4 T cell counts at w24 and with the expression of CD127 on T cells (Fig 4).

### Predictive factors for immune recovery

**Fig 5:**
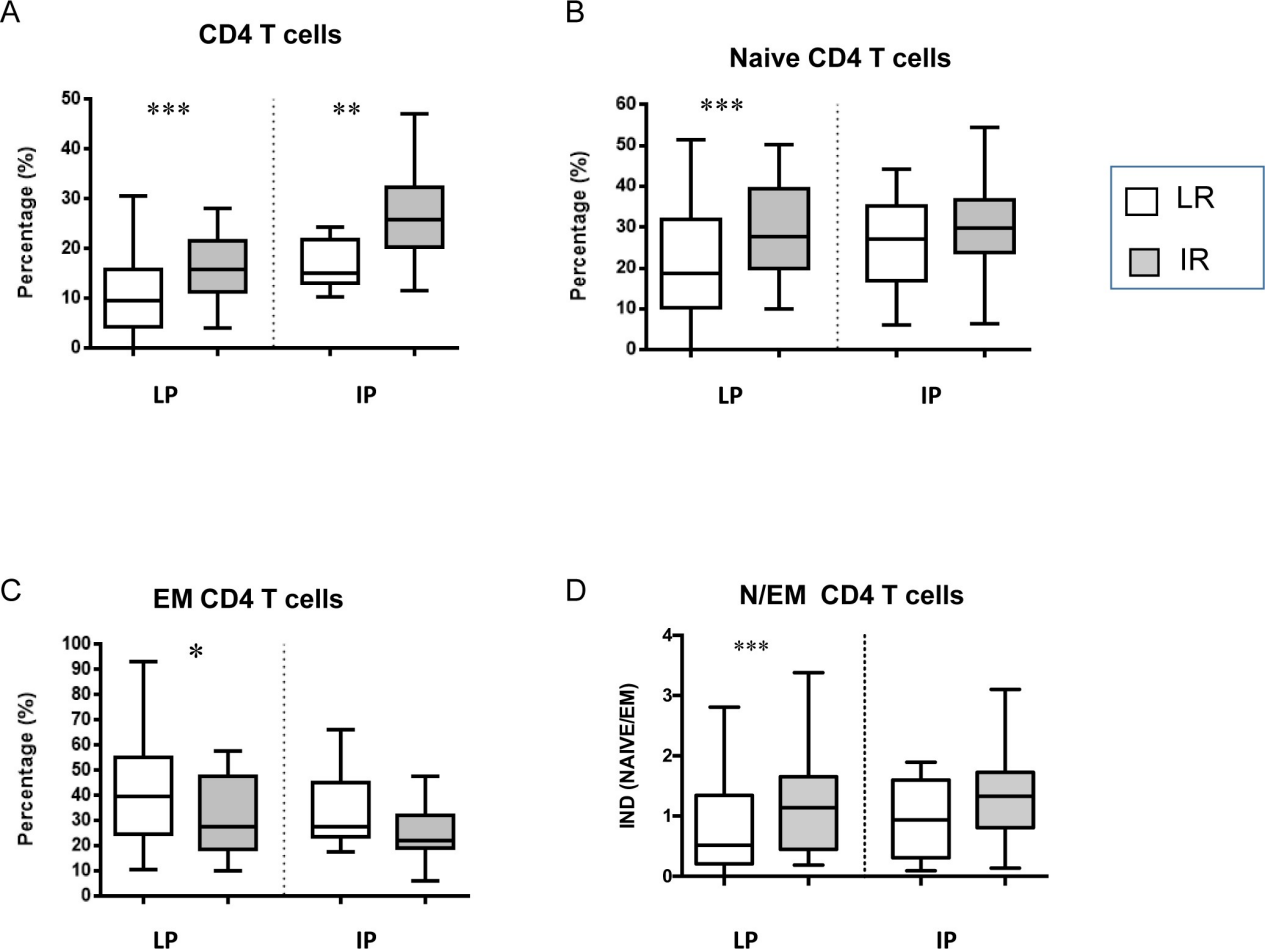
CD4 T cell differentiation was associated with immunological response to treatment. The frequency of total **(A)**, naïve **(B),** EM (**C**) CD4 T cells and the N/EM CD4 T cell ratio (**D**) was analyzed before (w0) and after (w24) treatment in immunological responders (IR, grey bars) and in immunological low responders (LR, white bars). Results are shown as Box and Whiskers graphs. The parametric Wilcoxon test was used and p<0.05 was considered significant.

To assess possible associations between immunological markers and optimal immune recovery (defined as the achievement of 500 CD4/mmc at w24), we focused on IP and LP patients. Thus, we compared optimal immunological responders (IR, HIV patients reaching 500 CD4 T cells/mmc at w24) and low immunological responder (LR, HIV patients with CD4 T cells at w24 lower than 500/mmc). Using this approach, the frequency of IR was 31.3% (31/99) in LP and 80.7% (42/52) in IP patients. To evaluate possible associations between specific immunological variables at baseline and immune recovery, we compared immunological T cell subsets at w0 in LR (Fig 5, white boxes) and in IR (Fig 5, grey boxes). The IR showed a significant higher CD4 T cell frequency at w0 when compared with LR in both HIV groups (Fig 3A). Moreover, in LP patients, a higher frequency of naïve and a lower frequency of EM CD4 T cells was observed in IR patients when compared to LR (Fig 5B and 5C). Finally, in LP, a higher naïve/effector CD4 T cell ratio was observed in IR respect to LR (Fig 3D).

We therefore evaluated the ability of immune markers to predict the immune recovery using a dependent variable, defined as the change of CD4 T cell absolute number between w0 and w24. We used N/EM CD4 T cell ratio as divided in 4 groups by quartiles (1° quartiles = 0.46, median = 1.14, 3° quartiles = 1.89). The variable N/EM CD4 T cell ratio was not significantly different in the four groups by univariate statistical analysis (p = 0.262). In contrast, a multivariate exploration, performed adjusting for age (< = 37 vs. >37), CD4 T cell count at w0 (<350 and > = 350) and CD4/CD8 T cell ratio at w0 (<0.30 vs. 0.30–0.45 vs >0.45) showed significant differences. Indeed, we found that subjects with a value of N/EM CD4 T cell ratio equal or higher to third quartiles showed an average recovery of 96 cells CD4 at w24 more than patients with N/EM CD4 T cell ratio value lower than the first quartile (Table 3).

**Table 3:** Multivariable linear regression analysis exploring the association with the dependent variable: change of CD4 T cell count from w0 to w24.

	Beta	95% CI	*p*
**Age**			
<37	Ref		
> 37	-6.59	-60.82 ; 47.64	*0*.*810*
**Baseline CD4**			
< 350	Ref		
> 350	-11.89	-34.90 ; 11.12	*0*.*309*
**CD4/CD8 ratio**			
< 0.30	Ref		
0.30–0.45	74.84	2.08 ; 147.61	*0*.*302*
> 0.45	55.61	-16.92 ; 128.13	*0*.*132*
**N/EM ratio**			
< 0.46 (I quart)	Ref		
0.46–1.14 (I-II quart)	10.83	-57.92 ; 79.58	*0*.*756*
1.14–1.89 (II-III quart)	42.97	-29.12 ; 115.06	*0*.*241*
>1.89 (III quart)	96.24	11.33 ; 181.15	***0*.*027***

## Discussion

This study describes the feasibility of a multicentric flow cytometric protocol for the analysis of CD4 and CD8 T cell homeostasis in HIV patients under 24 months of cART. Using this method, we showed the modulation of immunological markers by cART introduction, and we identified prognostic factors for early immune-recovery, useful in clinical management of late presenter HIV patients.

HIV infection strongly induces a perturbation of CD4 and CD8 T cell homeostasis. In untreated patients, T cells are activated, show a differentiation profile skewed toward effector cells and express markers of immune exhaustion [20]. cART introduction can reduce these immunological unbalances, but failed to completely restore a healthy immune system. It was clearly defined as early cART represents the best strategy to ensure a rapid immune reconstitution and to efficiently reduce the immune activation and the establishment of a persistent inflammatory environment [23]. Indeed, the time of cART initiation is a key factor determining and shaping the immune recovery, being an early treatment more effective in terms of immune preservation. Nevertheless, despite several strategies focused in achieving early diagnosis and treatment, a large proportion of HIV patients experienced a late diagnosis [24]. In these patients, the definition of markers able to predict and monitor the immune reconstitution potential of each patients would be highly useful for optimal clinical management.

After cART, a significant increase of CD4 and a parallel decrease of CD8 T cell frequency was observed in all groups of patients, independently from the CD4 T cell count before enrolment, resulting in an improvement of CD4/CD8 T cell ratio. Moreover, an increase of naïve T cells and a parallel reduction in EM T cells were observed in all patients, suggesting that cART can improve the differentiation profile [21], probably by clearing viral antigens and by reducing T cell activation. Indeed, according to previous observation [25], a reduction of CD38 on CD8 T cells was shown in all groups of HIV patients. In contrast, a significant reduction of CD95 and a parallel increase of CD127 on T cells was observed only in LP and in IP patients. HIV infection is known to destroy the homeostatic environment for T cells [26], inducing an increase of T cells activation (CD38, CD57, CD95 [27,28]) and a parallel reduction of healthy marker expression (CD127) that can be only partially reverted by treatment introduction [21,26]. CD127 expression on T cells is crucial for differentiation, survival and homeostatic proliferation potential of naïve T cells [29,30]. Reduced expression of CD127 on T cells has been associated with disease progression in HIV infected patients, can be reduced by effective cART treatment [31] and can impair the immune recovery [32]. We found that the baseline CD127 expression was negatively correlated with the frequencies of EM CD4 T cells, suggesting a reciprocal effect of skewed differentiation and reduction of T cell homeostatic potential in viremic HIV patients.

We showed that the frequency of naïve and EM CD4 T cells before treatment are strongly correlated with the expression of several other parameters associated with the outcome of HIV infection. Naïve CD4 T cell positively correlates with the expression of beneficial immune parameters (CD4, CD4/CD8 T cell ratio and CD8 naïve T cells) and negatively with markers of HIV disease progression (CD95, CD8, EM CD4 and CD8 T cells). EM CD4 T cells show an opposite correlation profile. Therefore, these data obtained from this multicentric study, suggest that naïve and EM CD4 T cell frequency could be used as easy markers mirroring several other immune features in untreated HIV patients, useful for the definition of the healthy immune system of each patient.

Moreover, the measurement of naïve T cells has been also suggested to predict the immune reconstitution in HIV infected patients [13,33]. Our data showed that immunological responders patients (defined as those achieved CD4 T cell count >500/mmc at w24) presented a higher frequency of naïve CD4 T cells and a lower frequency of effector T cells. Moreover, the immune reconstitution capability of LP was associated to a higher N/EM T cell ratio at w0, suggesting that this marker can be beneficial for the management of LP patients. Moreover, in LP and IP patients, the multivariate analysis revealed that N/EM T cell ratio higher than 1.89 is associated with a higher increase in CD4 T cell numbers after 24w months of therapy, confirming the feasibility of N/EM ratio as a predictor of immune reconstitution slope in patients with low CD4 T cells count. Several markers have been positively or negatively associated to the immune reconstitution such as the nadir CD4 T cells [7,8] CD4/CD8 T cell ratio [9–12], specific functional subset of T cells [18], inflammation [1] and gut microbiota [19]. These markers define complex pathogenetic processes undergoing during HIV infection, and represent critical issues to be better dissected mainly in patients with low CD4 T cell counts. Nevertheless, the clinical management of HIV disease needs easy, rapid and reliable markers able to depict the complex immunological profile of each patient and to predict their specific immune behavior after treatment initiation. Our data propose the N/EM CD4 T cell ratio as an index able to mirror the healthy immune system of each patient before treatment and to predict the immune reconstitution slope in the first months after cART initiation. A critical point about the introduction of new markers able to improve HIV management is to limit the increase in costs and, on the other hand, to allow its use also in middle-low income settings. The N/EM CD4 T cell index should be analyzed only in late presenter patients (which represents the main population at risk for a worse or delayed immune reconstitution), could be centralized in laboratories equipped with a 6 color flow cytometer by shipping blood samples at room temperature until 24 hours from the withdrawal.

The main limitation of this study was the short follow-up (24w, i.e. 6 months of treatment) that was initially defined in order to evaluate short term cART effect on immunological profile. Further studies are necessary to define the ability of naïve/EM T cell ratio in predicting long lasting immune recovery and co-morbidities occurrence in prospective cohorts, to verify its usefulness in every settings.

Altogether, our data define an easy protocol able to define reliable immunological markers useful for the characterization of immune profile in viremic HIV patients and identify the naïve/effector CD4 T cell ratio as a new tool able to early predict the immune reconstitution potential.

## Supporting information

S1 Database (XLSX)
